# Supplementary material for: Modeling and Visualization of Nitrogen and Chlorophyll in Greenhouse Solanum lycopersicum L. Leaves with Hyperspectral Imaging for Nitrogen Stress Diagnosis
Source: Plants (Basel). 2025 Oct 27;14(21):3276. doi: 10.3390/plants14213276 (PMC12608197; doi:10.3390/plants14213276)
Supplement: Supplementary file 1 [file plants-14-03276-s001.zip › plants-3923678-supplementary.pdf]

Table S1. The classification of wavelength sets for fine extraction by IRIV.

Wavelength selection algorithms	Strong information wavelength	Weak information wavelength	Wavelength of irrelevant information
iRF-CARS	689、690、767、780、786	434、459、461、463、479、480、481、488、489、503、507、508、509、510、707、712、786、787、789、790、803、816、824、826、831、835、840、845、863、870、871、875、879、886、894	457、819
iRF-BOSS	509、633、766、792	434、457、461、478、479、481、488、688、689、692、707、710、760、787、790、801、803、804、808、810、816、822、831、838、843、863、867、870、871、876、887	819
iRF-VCPA	689、766、779、870	488、502、509、633、787、789、803、894	-
iVISSA-CARS	435、488、689、690、766、787、788、789、790、803、876	436、442、453、459、478、481、489、499、502、503、506、508、509、510、550、552、591、621、637、642、659、662、687、691、712、767、768、771、772、779、780、781、793、797、808、813、816、831、838、870、871、872、875、879、886、894	452、505、511、611、615、616、791、792、895
iVISSA-BOSS	689、766、779	434、435、452、482、487、488、502、503、508、590、626、630、632、633、641、647、659、687、688、712、787、788、790、798、803、804、808、813、815、822、836、843、876、881	707
iVISSA-VCPA	766、780	435、488、502、509、642、662、767、787、803、870	-

Leaf segments (2 × 2 mm) were homogenized and 0.2 g of the fresh sample was used for chlorophyll content determination. The remaining leaves were placed in kraft paper bags and oven-dried at 105 °C for 30 minutes for enzyme deactivation, followed by drying at 80 °C for 72 hours until constant weight was achieved. The dried leaves were ground into fine powder using a laboratory grinder. Between 0.1000 and 0.2000 g of the powdered sample was weighed into a digestion tube, moistened with distilled water, and mixed with 5 mL of concentrated sulfuric acid. The tube was then heated gently in a digestion block. When white fumes appeared, the temperature was gradually increased until the solution turned brownish-black. The tube was briefly cooled, and 30% H_2O_2 was added dropwise with continuous shaking before reheating. This H_2O_2 addition was repeated 2–3 times until the digest became colorless or clear, followed by further heating

for 5–10 minutes to remove residual H_2O_2. The digest was subjected to semi-micro distillation for nitrogen determination, with a blank assay performed in parallel to correct for reagent impurities. A Kjeldahl nitrogen analyzer was used to distill the cooled digest, automatically adding sodium hydroxide and absorbing the released ammonia in boric acid solution. Finally, titration was carried out using a pre-prepared standard hydrochloric acid solution to quantify the total nitrogen content.

$$NC(\%) = \frac{14 \times (V_1 - V_0) \times C \times T_s}{10 \times m} \times 100\% \quad \text{(S1)}$$

Where, *NC* denotes the total nitrogen content (%). The constant 14 represents the molar mass of nitrogen (g/mol). V_1 is the volume of standard acid consumed in the sample titration (mL). V_0 is the volume of standard acid consumed in the blank titration (mL). *C* indicates the concentration of the standard acid (mol/L). T_s stands for the aliquot factor (value = 1 in this experiment). *m* refers to the mass of the dry sample weighed (g).

The 0.2 g of leaf segments for chlorophyll content determination was transferred into a test tube. First, 10 mL of 96% anhydrous ethanol was added, mixed by shaking, and the mixture was kept in the dark for 10 hours for extraction. Then, an additional 10 mL of 96% anhydrous ethanol was added, followed by shaking and further extraction in the dark for 14 hours. Finally, 5 mL of 96% anhydrous ethanol was added to bring the solution to volume. The absorbance of the prepared extract was measured at wavelengths of 665 nm, 649 nm, and 470 nm. Each sample was measured in triplicate, and the average value was used to calculate the chlorophyll content according to the following equation.

$$Chlac = 13.95 \times A_{665} - 6.88 \times A_{649} \quad \text{(S2)}$$

$$Chlbc = 24.96 \times A_{649} - 7.32 \times A_{665} \quad \text{(S3)}$$

$$Chlsc = Chla + Chlb \quad \text{(S4)}$$

Where, *Chlac*, *Chlbc*, and *Chlsc* denote the concentrations of chlorophyll a, chlorophyll b, and total chlorophyll, respectively (mg/L). A_{665}, A_{649}, and A_{470} represent the absorbance of the pigment extract at wavelengths of 665 nm, 649 nm, and 470 nm, respectively. Based on the mass of the leaf sample and the volume of the extraction solution, the chlorophyll concentration (mg/L) can be converted to chlorophyll content per unit leaf mass (mg/g) using the following conversion formula:

$$Chla = (Chlac \times V) / (1000 \times m) \quad \text{(S5)}$$

$$Chlb = (Chlbc \times V) / (1000 \times m) \quad \text{(S6)}$$

$$Chls = Chla + Chlb \quad \text{(S7)}$$

Where, *Chla*, *Chlb*, and *Chls* represent the contents of chlorophyll a, chlorophyll b, and total chlorophyll (mg/g), respectively. *V* denotes the volume of the extraction solution (25 mL in this experiment). *m* indicates the mass of the leaf sample (0.2 g in this experiment).
